# Anti-thrombotics and their impact on inpatient epistaxis management: a tertiary centre experience

**DOI:** 10.1007/s11845-021-02790-1

**Published:** 2021-09-25

**Authors:** Gavin Donaldson, Kwee Yen Goh, Puneet Tiwari, Sangeeta Maini, Bhaskar Ram, Raghav C. Dwivedi

**Affiliations:** 1 Department of ENT and Head & Neck Surgery, Aberdeen Royal Infirmary, Aberdeen, UK; 2 The School of Social Sciences, Nottingham Trent University, Nottingham, UK

**Keywords:** Anti-coagulants, Anti-platelets, Emergency, Epistaxis, Management

## Abstract

**Introduction:**

Epistaxis represents a massive burden upon NHS resources. Despite being an extremely common reason for emergency ENT admissions, there remains significant variation in its management. Although the evidence base is continually growing, there appears to be a lack of guidance towards managing anti-coagulants and anti-platelet medications and identifying patient-specific outcomes in this setting. Epistaxis has long been associated with a multitude of risk factors but none have shown consistent, direct correlation.

**Materials and methods:**

We aimed to identify if the use of anti-thrombotic medication was associated with a longer length of hospital admission or conferred a higher requirement for nasal packing, re-packing, surgery or re-admission. We conducted a retrospective analysis of 100 consecutive adult patients admitted over a 6-month period. Statistical analysis was conducted using SPSS software.

**Results:**

Sixty-five percent of patients were taking anti-thrombotic medication. The variability of admission INR values in those taking warfarin did not relate with any outcome measure. There was no statistical difference between patients taking anti-thrombotic medication and those who do not, with regards to our primary outcome measures. Re-admission rates within 28 days were found to be 13%, with anti-thrombotic medication use and pre-existing cardiovascular disease recognised as commonly encountered risk factors. Three percent of patients required surgical intervention. Eight percent of patients required re-packing, with a Rapid Rhino chosen in all instances.

**Conclusion:**

The use of anti-thrombotic medication is not associated with increased morbidity or increased rate of complications. Anti-thrombotic usage and more than one medical co-morbidity increase the risk of re-admission within 28 days.

## Introduction

Epistaxis represents the most common emergency admission to ENT departments nationwide, accounting for a third of all emergency admissions [1]. In the UK, epistaxis admissions result in approximately 39,000 bed days placing a significant burden on our healthcare system [2]. The true prevalence of epistaxis is largely underestimated as data has revealed that up to 60% of the UK population have experienced a nosebleed, with only 6% pursuing medical attention [3, 4]. Although epistaxis is not often fatal, it certainly represents a significant level of morbidity. The aetiology of epistaxis can be attributed to local or systemic factors; however, there is often a degree of overlap, and in fact, a cause may not be identified during medical assessment [5–7].

Specific patient demographics and risk factors for epistaxis are poorly understood with a paucity of evidence to determine a definitive causative relationship [8]. Despite the common finding of hypertension during the acute presentation of epistaxis, a systematic review could not conclude a direct causative relationship due to other confounding factors including stress and possible white coat phenomenon [8]. A study by Corte et al. [9], however, concluded that predictive factors of admission to the emergency department due to epistaxis were male gender, older age, peripheral vascular disease, cardiovascular disease and history of epistaxis.

Furthermore, re-presentation rates have been found to be in the region of 14% with recent studies identifying risk factors which may be influential in predicting recurrent episodes of epistaxis [10]. Sustained ambulatory hypertension and anti-coagulant or anti-platelet use appear to have the greatest association [11]. Conti and colleagues [12] demonstrated that four in 10 patients presenting with major bleeding in an ED department during a 2-year survey showed hypertension. However, this was not epistaxis-specific. They also concluded that patients taking warfarin compared with direct oral anti-coagulants (DOACs) were more likely to present with major bleeding [12]. We will endeavour to clarify if this finding is also seen in our study population.

With the introduction of DOACs, in conjunction with increasing polypharmacy, evidence has shown that this can increase the length of hospital stay, further increasing the cost and burden of inpatient stays [2, 13]. Furthermore, in the UK, the prescription of DOACs has now overtaken that of warfarin in new diagnoses of atrial fibrillation (AF) [14].

A recent UK multi-centre audit has demonstrated significant variation of inpatient management of epistaxis, subsequently resulting in the development of a multi-disciplinary consensus guideline [13]. Despite this, there remains a lack of high-quality evidence supporting specific areas often complicating clinical decision-making. Epistaxis-specific strategies for concurrent use of anti-thrombotic medications largely rely on expert opinion. The variability in management was highlighted by Villcock et al. [15] in 2013 suggesting the need for further studies to elucidate variables affecting outcomes in the various treatment options for epistaxis. Although national data has been collected in relation to inpatient epistaxis management, analysis of current practices and patient demographics from a Scottish perspective is lacking.

We aim to explore the current inpatient management strategies adopted within a tertiary Scottish ENT department with respect to managing those taking anti-thrombotic medication and particular patient outcomes. We are particularly interested in those aspects of management that culminate in higher rates of morbidity and increased length of stay.

## Materials and methods

This retrospective observational cohort study identified consecutive patients admitted with a primary diagnosis of epistaxis to a tertiary ENT department over a 6-month period, from January to June 2017.

### Inclusion and exclusion criteria

Patients aged 18 or over, presenting as emergency cases with a diagnosis of epistaxis (as defined on their discharge documentation and coded appropriately), were admitted to our ENT ward. This included referrals from local emergency departments, GP practices or any inpatient referrals. Patients reviewed at our rapid access ENT clinic who were not subsequently admitted were excluded. We were unable to record outcome measures of patients not requiring admission to our unit. Consequently, our study represents a sample of inpatients; therefore, generalising these findings should be done with caution.

### Data collection

Utilising data from the local clinical coding department in Aberdeen Royal Infirmary, patients were selected if their primary diagnosis on their final discharge letter was coded as epistaxis. This database of patients included further details of length of admission, medical and surgical interventions, use of anti-thrombotic medication, co-morbidities and results from any coagulation studies. We used this specific information for explorative analysis.

The pre-defined primary outcome measures included the identification of patients taking anti-thrombotic medication pre-admission and recording this effect on length of hospital admission, need for nasal packing and re-packing, need for surgical intervention and re-admission rates. We decided to group anti-coagulants and anti-platelets together as they are often considered a single risk factor within the literature despite representing completely different modes of action.

Secondary outcomes included the recording of INR values taken at the time of admission and identifying the effects of a therapeutic v non-therapeutic value.

### Statistical analysis strategy

Statistical analysis was performed using SPSS (Version 24). Variables were expressed as the mean ± standard deviation. Statistical comparisons were made between patients taking anti-thrombotic medication (*n* = 65) and those not (*n* = 35) pertaining to length of hospital admission (days), requirement for nasal packing or re-packing, requirement for surgical intervention and re-admission. Length of hospital stay was not normally distributed, as confirmed by Shapiro–Wilk’s test; therefore, non-parametric tests were used for analysis. Chi-squared tests were used to compare the categorical variables described.

The relationship between the admission INR value, in those patients taking warfarin, and the length of admission was analysed using the Mann–Whitney *U* test. The relationship between therapeutic levels of INR and patient outcomes was analysed using the chi-squared test of association for the pre-defined nominal variables. A *p* value of < 0.05 was considered statistically significant.

## Results

A total of 102 cases were identified over a 6-month period. Two cases were deemed unsuitable due to incomplete datasets. Ultimately, 100 patients fulfilling the inclusion criteria were included.

The median age of included patients was 73 (range = 24–93) years; interquartile range (IQR) = 17. Seven patients were aged 50 or below. Of these patients, none were taking any form of anti-coagulant medication and all were initially packed with a non-dissolvable nasal pack. The aetiological factors within this age group appear different from that of the older patient cohort and are outlined in Table 1. One of these seven patients required re-admission within the 28-day follow-up period.

**Table 1:** Frequency of aetiological factors seen in patients aged < 50 years old

Aetiology	Frequency
Post-traumatic	2
Post-operative (within 7 days)	2
Idiopathic	3

Of the 100 patients included, there was a considerable male predominance representing 69% of all patients with the remaining 31% representing female patients.

The average length of hospital stay within the whole study sample was 1.64 nights. In patients in whom any form of nasal packing was inserted, their average length of hospital stay was 2.12 nights compared with 1.00 night for those who were not packed.

### Anti-thrombotic medication usage and length of hospital admission

**Fig. 1:**
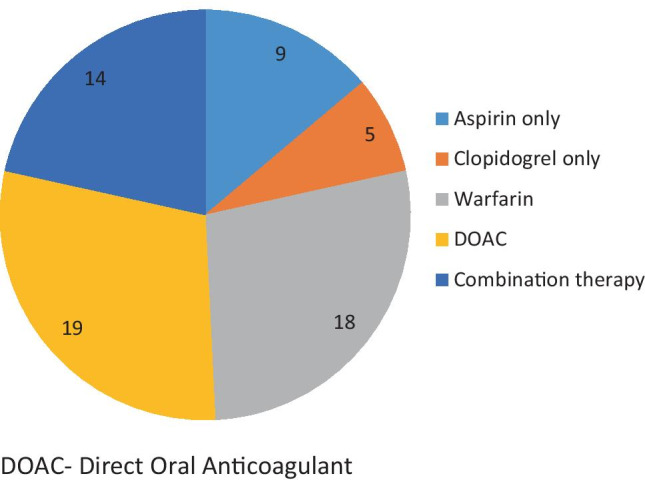
Number of patients taking each class of anti-thrombotic medication at the time of admission

In total, 65 patients were taking some form of anti-thrombotic medication. Figure 1 demonstrates a breakdown of the classes of anti-thrombotic medication encountered in this study.

The most common class of medication recorded were DOACs accounting for 19 (29.2%) cases, with 84% of these patients taking rivaroxaban and the remaining 16% taking apixaban. No patients admitted with epistaxis were prescribed any of the direct thrombin inhibitors, including dabigatran.

Patients not taking anti-thrombotic medication had a shorter mean length of hospital stay (1.42 v 1.73 nights); however, this exhibited no statistical difference utilising the non-parametric Mann–Whitney *U* test. We specifically analysed each subcategory of anti-thrombotic class against the no anti-thrombotic use group. No class of drug demonstrated any statistically significant difference in length of stay.

### Warfarin and effect of INR values

Eighteen patients were taking warfarin. At admission, INR values were measured in 16/18 patients. The mean INR value was 1.98 (range 1.8–3.8). Only one patient had a higher target INR range of 2.5–3.5 for a prosthetic aortic valve, and in this case, the INR was 3.7. The most common indication for warfarin therapy was AF accounting for 15 (83%) of the cases.

Of the patients taking warfarin, seven (43.7%) patients’ INR were within the therapeutic range whilst five (31.3%) had an elevated and four (25.0%) had a lower value compared to their documented desired range. Table 2 outlines how the INR level affected particular patient outcomes.

**Table 2:** Outcomes of patients taking warfarin according to admission INR levels

INR classification	Cases (*n*)	Duration of admission (average nights)	Patients requiring nasal packing	Patients requiring re-packing	Patients requiring surgical intervention	Patients re-admitted with epistaxis
Normal	7	7 (1.0)	5	0	0	0
Supra-therapeutic	5	6 (1.2)	5	1	0	0
Sub-therapeutic	4	9 (2.25)	4	0	0	0
Not performed	2	0 (0.0)	0	0	0	1
Statistical analysis of normal v abnormal INR (Pearson’s chi-squared value unless specified)	N/A	Mann–Whitney *U* test = 0.379	0.900	0.650	0.745	0.241

*INR* international normalised ratio

A specific comparison was made between patients with a normal and an abnormal INR value (including both supra- and sub-therapeutic values). There was no statistical difference between a normal and an abnormal INR in length of stay, as seen from the Mann–Whitney *U* test (*p* value = 0.379).

Additionally, there was no statistical difference with regards to the requirement for surgical intervention and re-admission rates. Therapeutic levels of INR and the need for nasal packing were weakly related. This association was depicted by Kramer’s *V* value of 0.219.

### Anti-platelet medications

A total of 14 (21.6%) patients were taking anti-platelet medications pre-admission, nine (64.3%) taking aspirin and five (35.7%) taking clopidogrel. With such small numbers, we were unable to identify any statistical differences in outcomes for these two groups; however, we did observe that those taking aspirin had a longer length of hospital stay compared to the entire sample (2.0 v 1.2 nights).

### Combination therapy

Fourteen patients (21.6%) were taking a combination of anti-thrombotic medication. The most common combination seen in our cohort was aspirin and clopidogrel which was prescribed for secondary cardiovascular prevention strategies in all cases. The exact combinations seen are outlined in Table 3 along with their relationship with average length of hospital stay, requirement for nasal packing and re-admission rates. No patients taking combination therapy required surgery or re-packing.

**Table 3:** Outcome measures according to specific combination anti-thrombotic therapy

Combination therapy	Cases (*n*)	Indication for combination therapy	Average length of hospital stay	Number of patients requiring nasal packing	Patients requiring re-admission
Aspirin & clopidogrel	8	Secondary cardiovascular prevention	2.5	6	2
Warfarin, aspirin & clopidogrel	1	Mechanical heart valve	1.0	1	0
Warfarin & clopidogrel	2	Metallic mitral valve & post-PCI	1.5	1	0
Ticagrelor & aspirin	1	Post-ACS	0	0	0
Apixaban, aspirin & ticagrelor	2	Post-ACS	2.5	1	2

*PCI* primary coronary intervention

*ACS* acute coronary syndrome

Details of anti-thrombotic medications and their association with our four pre-defined primary outcome measures are shown in Table 4.

**Table 4:** Inpatient details of patients depending on their use of anti-thrombotic medication

Medication	Cases (*n*)	Average length of hospital stay (nights)	Number of patients requiring nasal packing (%)	Number of patients requiring re-packing	Need for surgical intervention	Patients requiring re-admission
Aspirin	8	2.0	4 (50)	0	1	1
Clopidogrel	5	1.22	2 (40)	1	1	0
Warfarin	18	1.22	14 (78)	1	0	1
DOACs	19	1.84	15 (79)	1	0	1
Combination therapy	14	2.07	9 (64)	1	0	4
No anti-coagulants	35	1.42	25 (71)	4	1	6
Statistical analysis of anti-thrombotic use v no anti-thrombotic use groups (Pearson’s chi-squared value unless specified)	N/A	Mann–Whitney *U* test = 0.808	0.928	0.930	0.951	0.366

### Surgical intervention

Three (3%) patients required surgical intervention, two underwent sphenopalatine artery ligation and one underwent a combined sphenopalatine and anterior ethmoidal artery ligation. All three patients were male with a mean average age of 63. Two of the three patients were taking prophylactic doses of anti-platelet medication. None of these patients required re-admission within 28 days of discharge.

### Nasal re-packing

Eight patients (8%) required their nasal pack to be replaced during their admission. We were unable to reliably identify the reason in each case. Table 5 demonstrates the specific case details of these eight patients according to which pack was initially inserted.

**Table 5:** Specific details of patients requiring re-packing

Type of initial pack inserted	Cases (*n*)	Total number of nights in hospital	Average length of hospital stay (nights)	Patients taking anti-thrombotic medication	Need for surgical intervention?	Patients requiring re-admission
Rapid Rhino	4	18	4.5	2/4	No	2
Merocel	3	5	1.67	2/3	No	0
Nasopore	1	3	3	0	No	0

Of the eight patients requiring re-packing, seven were male and had a mean average age of 70 years. These patients remained in the hospital for an average of 3.25 nights. Half of these patients were taking anti-thrombotic medication, with only one patient taking warfarin. This patient was found to have an elevated INR of 3.4 at the time of admission. Two of these patients were re-admitted within 28 days and none required any form of surgical intervention. In all cases, the replacement nasal packing of choice was a Rapid Rhino. No patients required re-packing more than once.

### Re-admission rates

A total of 13 (13%) patients required re-admission within 28 days of discharge. The demographics and inpatient details are outlined in Table 6.

**Table 6:** Risk factors identified in patients requiring re-admission

Risk factor	Value/number of patients (from a total of 13 patients requiring re-admission)
Average age	62
Male: female ratio	9:4
Occurrence of bilateral epistaxis	6
Required nasal packing during index admission	9
Use of anti-thrombotic medication	7
Surgical intervention (during either admission)	0
Identifiable cause (other than idiopathic)	8

## Discussion

Our retrospective study aimed to identify trends in our current practice of managing inpatient epistaxis patients. This study represents one of the largest retrospective reviews across Scotland, and in light of the variation identified in the recent UK-wide multi-centre audit, this will add meaningful data specific to a Scottish population [10].

The average length of stay in our 100-patient cohort was 1.64 days, longer than that found in the 2016 UK audit of inpatient epistaxis of 1.23 days [10]. Although the no anti-thrombotic usage group had a shorter length of hospital admission, this exhibited no statistical difference. This finding is reiterated by Goljo et al. [16] and Sauter et al. [17] who found anti-coagulant usage was associated with a shorter length of hospital stay. Contrary to this, numerous studies have demonstrated that anti-coagulation use does in fact result in longer hospital admission [10, 11, 13, 14]. No specific anti-thrombotic class was associated with a shorter admission period compared to the no anti-thrombotic use group. Due to the department’s geographical location, patients travelling from long distances, including offshore islands, to be reviewed, are often admitted secondary to social reasons as opposed to a clinical decision. Furthermore, if we consider an elective ENT, a patient will require, on average, a 2-day admission; emergency admissions due to epistaxis occupy beds of approximately 700 elective admission per year [18]. This should encourage appropriate but efficient management of these patients.

The average age of patients was 73 with a range from 24 to 93. This represents a similar value to other large studies including the INTEGRATE audit of 1122 patients [10], averaging 73 years but higher than studies in other UK sites in Worthing, mean average 68.2 [19] years and a 6-site review in England, averaging 64.7 years [20]. Our average age is higher than that published in other countries including a study in Porto, Portugal, averaging 66 years [21] and 64.7 years recorded in a 2-year review of epistaxis in New York [15].

All but seven patients were aged over 50, suggesting that despite affecting all age groups, epistaxis predominantly affects an older population. This is supported by National Health Service (NHS) Hospital Episode Statistics for England between 2011 and 2012, with over 49% of adults admitted with epistaxis over the age of 75 [22]. Furthermore, this subgroup also had a propensity to have an identifiable cause of epistaxis as outlined in Table 1.

### Use of anti-thrombotic medication

In total, 65 patients (65.0%) were taking at least one form of anti-thrombotic medication, which is notably higher than the 51.0% quoted by the recent UK-wide national audit of inpatient epistaxis management in 113 UK hospital departments [10]. This may support the recent study by Douglas et al. [23] in Glasgow linking the incidence of cardiovascular disease with higher rates of socio-economic deprivation and thus requirement for secondary prevention therapies. The wide variation of anti-thrombotic use in England was highlighted by Hall et al. [20] in 2015, ranging between 35 and 70% of patients across six independent sites. These findings may reflect that patients admitted with epistaxis in our region tend to have more cardiovascular co-morbidities requiring medical treatment. Nonetheless, this should be considered when interpreting our results, as the use of anti-thrombotic medication has been shown to cause recurrent and heavier bleeding and an increased incidence of blood transfusion [11].

### Warfarin and the effect of INR values

**Fig. 2:**
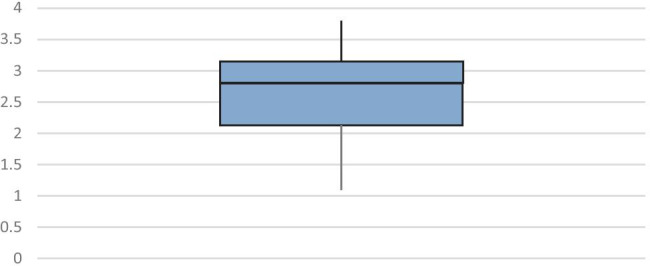
Box and whisker diagram showing the distribution of INR values in patients taking warfarin

We identified no direct correlation between the admission INR value of those patients taking warfarin and the subsequent need for nasal packing, surgery or re-admission. Furthermore, patients taking warfarin presenting with epistaxis were found to have a varied range to their admission INR value as depicted in Fig. 2. The length of stay was shortest in the group with a normal INR (1.0 night) with a longer duration seen in the sub-therapeutic (2.25 nights) and supra-therapeutic groups (1.2 nights).

Overall, 78% of patients taking warfarin required nasal packing. This observation highlights a propensity for patients taking warfarin therapy to require further interventions regardless of any first aid measures. Whether this trend accurately reflects the severity of bleeding or a tendency of medical staff to manage these patients with more definitive measures is debatable. The half-life of warfarin is 36–42 h; we therefore cannot definitely correlate the INR value at the time of admission with the anti-coagulant effect of this medication [24]. Ultimately, we have to manage such patients with the objective tests available at the time of their admission.

Despite the perception that warfarin, and in particular, an elevated INR, confers a higher risk of bleeding [25, 26], we have shown that this association may not be considered linear. Furthermore, a systematic review examining haematological factors, including warfarin, in the management of epistaxis could only identify a single interventional study concluding no significant difference in mean length of stay between the warfarin and control groups [27]. Our study would suggest basing guidelines solely upon admission INR values would not be recommended and other patient and surgical factors must be considered.

### Effects of different classes of AC meds

The number of patients taking either warfarin (18 patients) or a DOAC (19 patients) was similar. After comparing the outcomes of these two commonly encountered patient groups, it appears that both have similar rates of nasal packing, re-packing and re-admission. Interestingly, more patients were taking DOACs compared to warfarin alone (19 v 18), which seems to reflect data from Loo et al. [14], demonstrating that since 2015 DOACs have surpassed warfarin as the anti-coagulant of choice in patients newly diagnosed with AF in the UK [14]. If this trend is found to continue, we will have to continually update our knowledge and skills in this ever-changing field of anti-coagulated patients.

With regards to anti-platelet agents, observational studies have demonstrated an association between both the frequency and severity of epistaxis [28–30]. We analysed two specific classes: aspirin and clopidogrel, demonstrating no difference in patient outcomes.

Our study specifically analysed those patients taking combination anti-thrombotic therapy, a subgroup that poses a considerable challenge, but is often overlooked in other studies. Our data shows that dual anti-platelet therapy with aspirin and clopidogrel was the most common combination and this was associated with an increased length of hospital stay and also that three-quarters of this group required nasal packing and one-quarter were subsequently re-admitted. Although our results would suggest increased morbidity, there were no statistical differences in any outcome compared to the no anti-thrombotic use group.

Although the majority of patients admitted were taking at least one anti-thrombotic medication, a considerable proportion of patients (35%) were admitted without the presence of this risk factor. In fact, this subgroup of patients was found to have similar rates of nasal packing and re-packing when compared with those taking anti-thrombotic drugs. Moreover, we have shown that they also had a higher rate of re-admission (6 out of 35 patients). These findings highlight that anti-thrombotic use on its own is not the only risk factor which should be considered in patients presenting with epistaxis. Of course, we appreciate there is a multitude of other patient factors that can impact on rates of re-admission as described in Table 6.

### Surgery and re-packing rates

Due to the relatively small numbers of patients requiring surgical intervention, we could not conclude that any risk factors appear to favour this treatment modality. This is consistent with current literature which showed the early warning score did not differ between surgery and non-surgery groups [10]. The rate of surgical intervention of 3.0% was similar to that found in the INTEGRATE study (3.2%).^9^ We identified no patients requiring re-admission following surgery, this is contrary to other UK data reporting re-presentation rates of 22.6% [10]. Our data would suggest that surgery in the appropriate patient group might confer a favourable outcome in terms of re-admission rates. It must be noted that no patient underwent any form of interventional radiology procedure within our institution.

### Re-admission rates

Patients re-presenting with epistaxis after being recently discharged add an additional degree of complexity to our management decisions. We have identified that the use of anti-thrombotic medication and pre-existing cardiovascular disease are risk factors present in more than half of our patients. Patients with any underlying cardiovascular disease are often taking anti-coagulants as primary or secondary prevention; however, there were three patients who had at least one cardiovascular risk factor but were not taking any of these medications.

Our results were marginally better when compared with the INTEGRATE audit which identified re-presentation rates of 13.9% [10]. Further explorative analysis of re-admitted patients revealed a lower average age compared to the overall average (62 years v 71 years). This finding may suggest that the presence of medical co-morbidities rather than age alone is more predictive of re-admission rates. In keeping with national data, we have also shown that males are more likely to be admitted with epistaxis and have a higher tendency to require re-admission compared to their female counterparts.

## Limitations

This retrospective cohort study represents a relatively small number of patients admitted with epistaxis; therefore, the results should be interpreted as such. We were able to identify consecutive patients over a 6-month period and thus feel this patient sample is representative of our Scottish population. We also understand the multi-factorial nature of epistaxis and the many factors which must be considered when grading its severity and deciding upon the modality of treatment.

## Conclusion

Epistaxis is a multi-factorial disease with numerous patient and surgical factors involved in predicting the severity of bleeding. Our study suggests that the use of anti-thrombotic medication in patients admitted with epistaxis is not associated with an increased length of admission, nasal packing rates or requirement for surgery. Therefore, concomitant use of these medications should not be seen as a pre-requisite to inpatient management. The INR value recorded in those patients taking warfarin is not associated with our patient-specific outcomes. We have been able to identify two predominant risk factors in epistaxis patients that appear to be associated with higher rates of re-admission within 28 days: the concurrent use of anti-thrombotic medication and the presence of more than one medical co-morbidity. These factors should be clearly documented during assessment and be carefully considered when deciding upon hospital admission. This information should be used to guide further recommendations for the management of anti-thrombotic agents in epistaxis.

## Data Availability

Full transparency.
